# Expanded Newborn Screening in Italy: The First Report of Lombardy Region

**DOI:** 10.3390/ijns11020031

**Published:** 2025-04-25

**Authors:** Clarissa Berardo, Alessandra Vasco, Alessia Mauri, Simona Lucchi, Laura Cappelletti, Laura Saielli, Manuela Rizzetto, Davide Biganzoli, Cristina Montrasio, Diana Postorivo, Michela Perrone Donnorso, Elisa Pratiffi, Andrea Meta, Stephana Carelli, Alessandro Amorosi, Sabrina Paci, Graziella Cefalo, Francesca Furlan, Francesca Menni, Serena Gasperini, Viola Crescitelli, Giuseppe Banderali, Gianvincenzo Zuccotti, Luisella Alberti, Cristina Cereda

**Affiliations:** 1 Pediatric Research Center “Romeo ed Enrica Invernizzi”, Department of Biomedical and Clinical Sciences, University of Milan, 20157 Milan, Italy; clarissa.berardo@unimi.it (C.B.); alessia.mauri@unimi.it (A.M.); stephana.carelli@guest.unimi.it (S.C.); gianvincenzo.zuccotti@unimi.it (G.Z.); 2 Center of Functional Genomics and Rare Diseases, Department of Pediatrics, Buzzi Children’s Hospital, 20154 Milan, Italy; alessandra.vasco@asst-fbf-sacco.it (A.V.); simona.lucchi@asst-fbf-sacco.it (S.L.); cappelletti.laura@asst-fbf-sacco.it (L.C.); laura.saielli@asst-fbf-sacco.it (L.S.); manuela.rizzetto@asst-fbf-sacco.it (M.R.); davide.biganzoli@unimi.it (D.B.); cristina.montrasio@asst-fbf-sacco.it (C.M.); diana.postorivo@asst-fbf-sacco.it (D.P.); michelaperronedonnorso@gaslini.org (M.P.D.); elisa.pratiffi@asst-fbf-sacco.it (E.P.); andrea.meta@asst-fbf-sacco.it (A.M.); luisella.alberti@asst-fbf-sacco.it (L.A.); 3 Welfare General Directorate, Regione Lombardia, Palazzo Lombardia, Piazza Città di Lombardia, 1, 20124 Milan, Italy; alessandro_amorosi@regione.lombardia.it; 4 Clinical Department of Pediatrics, ASST Santi Paolo e Carlo Hospital, University of Milan, 20142 Milan, Italy; sabrina.paci@asst-santipaolocarlo.it (S.P.); graziella.cefalo@asst-santipaolocarlo.it (G.C.); giuseppe.banderali@asst-santipaolocarlo.it (G.B.); 5 Pediatric Intensive Care Unit, Fondazione IRCCS Cà Granda Ospedale Maggiore Policlinico, 20122 Milan, Italy; francesca.furlan@policlinico.mi.it (F.F.); francesca.menni@policlinico.mi.it (F.M.); 6 Pediatric Rare Diseases Unit, Fondazione IRCCS San Gerardo dei Tintori, 20900 Monza, Italy; serena.gasperini@irccs-sangerardo.it (S.G.); viola.crescitelli@irccs-sangerardo.it (V.C.); 7 Department of Pediatrics, Buzzi Children’s Hospital, 20154 Milan, Italy

**Keywords:** newborn screening (NBS), inborn errors of metabolism (IEMs), rare diseases, aminoacidemias (AAs), fatty acid oxidation disorders (FAODs), organic acidemias (OAs), urea cycle defects (UCDs), mass spectrometry (MS)

## Abstract

Background: Newborn screening (NBS) is a preventive healthcare program aiming at identifying the inborn errors of metabolism (IEMs) in asymptomatic infants to reduce the risk of severe complications. The aim of this study was to report the first years (2016–2020) of the expanded NBS program in the Lombardy region, Italy. Methods: Dried blood spots were collected from newborns’ heels at 48–72 h after birth. FIA-MS/MS was performed to evaluate specific biochemical markers. Genetic confirmation was achieved via Sanger or NGS on newborns and reported to a clinical reference center (CRC). Results: A total of 343,507 newborns were tested; 1414/343,507 resulted as positive to NBS and were reported to the CRC. A total of 209 newborns were diagnosed with IEMs: 206 infants received a diagnosis of IEM through NBS, confirmed by genetic analysis; three neonates were not positive to NBS but were subsequentially diagnosed with IEMs. A total of 1208/343,507 were false positive cases. Twenty-seven types of IEMs were diagnosed in 209 patients: 111 newborns were affected by aminoacidemias, 11 by urea cycle disorders, 27 by organic acidemias, 34 by fatty acid oxidation disorders, and 26 by secondary conditions. Conclusions: We report here for the first time the IEM incidence and distribution in the Lombardy region in the first five years of NBS.

## 1. Introduction

Newborn screening (NBS) is an important public health program developed for the early identification and treatment of actionable metabolic conditions as early as possible, so that appropriate treatments can be initiated promptly, preventing or minimizing the long-term complications [1,2].

Inborn errors of metabolism (IEMs) represent a group of genetic disorders resulting from alterations in the metabolic pathways responsible for processing various substances, such as amino acids, organic acids, fatty acids, glycogen, sphingolipids, coenzymes, and vitamins [3]. The defect results in either an increase or reduction in the concentration of these molecules that may lead to an accumulation of toxic substrates and their metabolites, deficiency of a reaction product, or insufficient provision of cellular energy [4]. The common acute clinical signs shared by most of the IEMs include vomiting, coma, and liver failure, while the chronic manifestations comprehend developmental delay, cardiomyopathy, and neurologic signs [5]. IEMs screened in Italy are classified as follows: aminoacidemias (AAs), urea cycle defects (UCDs), organic acidemias (OAs), and fatty acid oxidation disorders (FAODs) (Ministerial Decree of 13th October, https://www.gazzettaufficiale.it/eli/id/2016/11/15/16A08059/sg, accessed on 20 January 2025). Taken alone IEMs are rare; however, globally, they affect about 0.5–1 in 1000 people [3]. However, other disorders have been identified over time as disorders of glycogen storage, lysosomal storage, mitochondria, peroxisomes, and neuromuscular diseases [6].

While NBS is universally recognized as a life-saving practice, the structure and scope of NBS programs vary significantly. Besides the fact that they should fit Wilson’s and Jungner’s criteria [7], there is no consensus on the selection of diseases for NBS, which can differ between countries. Italy includes a greater number of screened disorders when compared with other European countries [8]. The NBS program in Italy is organized by the Ministry of Health and is implemented by the regional health authorities with 19 laboratories that perform extended NBS, which has been mandatory since 2016 (Ministerial Decree of 13th October, https://www.gazzettaufficiale.it/eli/id/2016/11/15/16A08059/sg, accessed on 20 January 2025). Lombardy is the region with the highest number of newborns screened, and the mandatory panel of IEMs screened by MS/MS is divided into the four main categories mentioned above.

Here, we present five years of NBS results in Lombardy, during which 343,507 newborns were screened between 2016 and 2020, with 209 patients diagnosed with a rare IEM. Since screening programs vary worldwide, and few Italian data are reported, this work is novel as it is the first one to present data on the incidence and distribution of IEMs in the Lombardy region.

## 2. Materials and Methods

### 2.1. Data Collection

In total, 343,507 newborns were screened in Lombardy, from 1 January 2016 to 31 December 2020. Written informed consent was obtained from all the newborns’ parents or legal guardians.

Dried blood spots (DBSs) were collected using the standard heel prick method between 48 and 72 h after birth in about 65 birth centers and 25 intensive care units. The DBSs were then sent to the Laboratory of Newborn Screening and Metabolic Rare Diseases at V. Buzzi’s Children’s Hospital for analysis. In preterm infants (gestational weeks <37 and >34), blood sample collection was repeated at approximately 14 days of life according to specific protocols. In the case of low-birth-weight infants (<2000 g) and severe preterm infants (<34 gestational weeks) blood sample collection was repeated twice, at 14 days and 28 days of life. NBS positive cases were referred to 3 Clinical Reference Centers (CRCs).

### 2.2. Panel of Screened Conditions

The mandatory panel was focused on the 45 diseases screened via MS/MS (Table 1). IEMs were classified into the four main groups mentioned above. A supplementary set of secondary conditions (SCs) was also considered in differential diagnosis, as they share the same primary markers.

### 2.3. DBS Analysis

The DBSs were processed using the Neobase non-derivatized newborn screening kit (PerkinElmer, Milan, Italy). Single 3.2 mm disks were punched using a DBS puncher (PerkinElmer, Italy) into 96-well plates. A total of 100 μL of the PerkinElmer extraction working solution was added to each well. The microplate was shaken for 45 min at 650 rpm at 45 °C and incubated for 120 min at room temperature to ensure the complete derivatization of the extracted succinylacetone. The plate was then quantified using a Waters Xevo TQD (Waters Corporation, Sesto San Giovanni, Italy) or Quattro Micro mass spectrometer in FIA-MS/MS. The concentrations were calculated by comparing the measured analyte intensities to those of the internal standards multiplied by the internal standard concentration and relative response factor. Eleven amino acids were analyzed: alanine (Ala), citrulline (Cit), arginine (Arg), glycine (Gly), leucine/isoleucine (Leu/lle/Pro-OH), methionine (Met), ornithine (Orn), phenylalanine (Phe), tyrosine (Tyr), and valine (Val). Thirty-one acylcarnitines were analyzed: C0, C2, C3, C3DC, C4, C4DC, C5, C5:1, C5DC, C5OH, C6, C6DC, C8, C8:1, C10, C10:1, C10:2, C12, C12:1, C14, C14:1, C14:2, C14OH, C16, C16:1, C16OH, C16:1, C18, C18:1, C18:2, C18OH, and C18:1OH.

The cut-offs reported in Table 2 were updated in 2018 from a reference population of 134,000 newborn samples >37 gestational weeks obtained at 48–72 h of life [9]. The first test was considered non-negative if one of the analytes resulted over/under the cut-off.

Based on the relative risk suspected according to the use of specific tools (see Section 2.6), newborns can follow different workflows, as described below.

The high-risk neonates were subjected to a retest and the 2TT, if available, on the same DBS. If the retest/2TT highlighted a high risk, the newborns were immediately referred to CRCs. If the retest did not confirm a high risk, the newborns were recalled for the performing of a second DBS (and/or a plasma or urine test) within 7 days.

**Figure 1:**
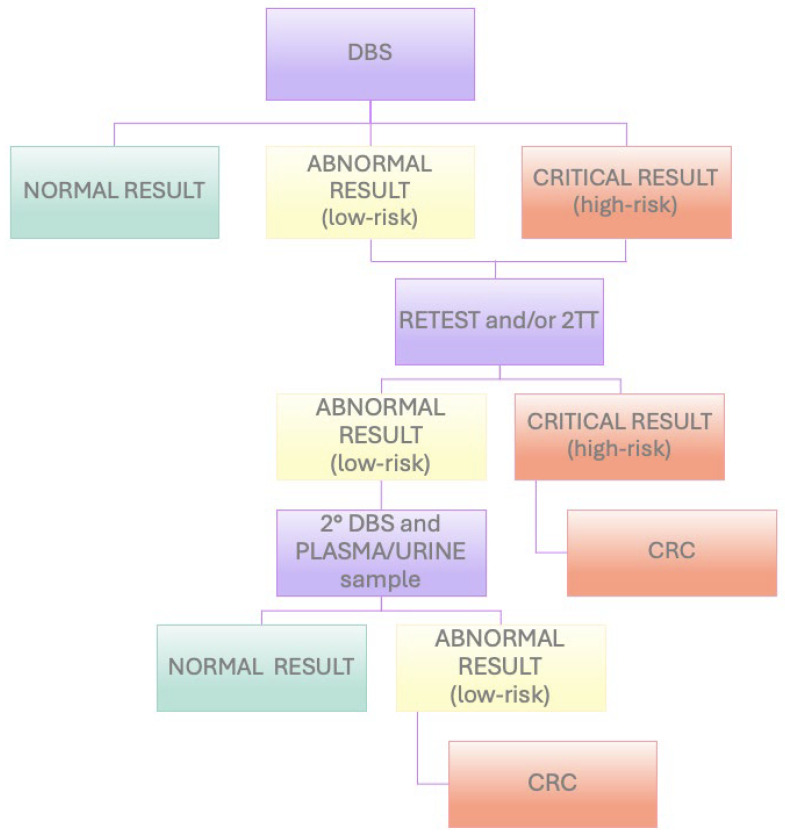
Flowchart of NBS workflow in Lombardy region (Italy).

The low-risk newborns with abnormal biochemical results were subjected to a retest/2TT on the same DBS, and if the alteration was confirmed, they were recalled for the performing of a second DBS (and/or plasma or urine test) within 7 days. If the altered result persisted in the second sample, the neonate was referred to CRCs (Figure 1).

#### Second-Tier Tests

Since 2016, 2TTs have been used with the quantitative dosage of methylmalonic acid, and then further 2TTs were gradually introduced and implemented, using a custom-made method. The DBSs were punched in duplicate and extracted using 100 µL of an aqueous solution of 0.1% formic acid, containing deuterated internal standards and tris(2-carboxyethyl)phosphine at specific concentrations. The plate was incubated at 37 °C for 25 min with agitation at 500 rpm. The supernatant was transferred to a new plate, which was sealed and analyzed via ultra-performance liquid chromatography-tandem mass spectrometry (UHPLC-MS/MS) on a Triple Quad 6500+ (SCIEX, Milan, Italy).

The 2TT result confirms or overrules the primary screening result. The use of the 2TT can identify the same target as primary screening but with improved specificity due to the separation from isomers or interfering substances, or it can screen for another diagnostic marker not included in the first-tier screening [10]. Patent: Perrone Donnorso, M., Cassanello, M., Cereda, C. A method and a kit for multiplexed 2nd tier test application in Newborn Screening. Pending Patent application n. 102024000022824, 14 October 2024.

### 2.4. Biochemical Confirmation Testing

The biochemical confirmation tests included the measurement of acylcarnitines (plACs) and amino acids (plAAs) in plasma samples as well as organic acids in urine.

For the plAC analysis, the samples were collected in Li-heparin and centrifuged at 3000 rpm for 5 min. A total of 3.2 μL of plasma was aliquoted onto a single 3.2 mm blank spot and then extracted and analyzed in the same way as for the NBS analysis.

The plAA measurement was determined via Ion-Exchange Chromatography with post-column ninhydrine derivatization. Blood samples in Li-heparin were collected, centrifuged, and deproteinized, and 50 µL of the sample was injected into the HPLC Amino acids Analyzer 30 PLUS (Biochrom US, Holliston, MA, USA). The samples were post-column derivatized u ninhydrin and were detected spectrophotometrically at a wavelength of 570 nm for all the amino acids, except for proline and hydroxproline detected at 440 nm.

Urinary organic acids were determined via gas chromatography–mass spectrometry (GC-MS) on the CP-3800 GC (Varian, Milan, Italy). After the extraction and derivatization, urinary samples were separated using programmed temperature capillary GC, and organic acids were identified from their electron impact mass spectra and retention time via comparison with internal and commercial mass spectral libraries.

### 2.5. Genetic Confirmation Testing

Genomic DNA was extracted from peripheral blood samples from positive newborns using the standard procedures. In the early years of NBS, sequencing was performed using Sanger technology; subsequently, NGS panel sequencing was employed.

Each variation is filtered based on the coverage (<10×), and gnomAD frequency, and evaluated in terms of effect (exonic and splicing) and phenotypes. Several in silico prediction tools (e.g., SIFT, PolyPhen2, Mutation Taster) were used to assess the pathogenic score of the identified variants, and disease-variant databases (e.g., HGMD, ClinVar, OMIM) were interrogated to assess the causative associations. The identified variant was interpreted according to the American College of Medical Genetics and Genomics guidelines with the support of several tools such as Varsome, Franklin by Genoox (https://franklin.genoox.com, accessed on 20 January 2025).

### 2.6. Post-Analytical Tools

The selection of a non-negative sample was performed using the Region 4 Stork tool which is an international collaborative NBS database that provides a score, using specific disease intervals for all the informative analytes, allowing a reduction in false positive rates and improved screening performance [11]. This tool was then substituted with the advanced version, “Collaborative Laboratory Integrated Reports” (CLIRs), which incorporates additional demographic information such as age, birth weight, and gender, using an algorithm (CLIR single condition tool) that determines whether a condition is Possible (low-risk), Likely, or Very Likely (high-risk) [12].

However, the presence of concerning symptoms, even in cases within the low-risk category, prompts a recommendation for testing and, when necessary, consultation with an appropriate specialist. No algorithm can account for all the possible scenarios or replace professional judgment.

## 3. Results

We examined the NBS data collected in Lombardy from 1 January 2016 to 31 December 2020. A total of 386,431 neonates were born, and 343,507 DBSs were evaluated for the detection of 44 IEMs.

### 3.1. Total Population NBS Data

A total of 1414/343,507 newborns (0.41%) resulted as positive to NBS and were referred to CRCs. A total of 209/343,507 (0.06%) newborns were diagnosed with IEMs: 206/343,507 infants (0.06%) received a diagnosis of IEMs through the NBS program, confirmed via genetic analysis and started a specific treatment and follow-up to CRCs; 3/343,507 neonates (0.0009%) were not referred to the CRCs through the NBS program but were subsequently diagnosed with IEMs; therefore, they were considered as NBS false negatives. A total of 1208/343,507 newborns (0.35%), which corresponded to 85.43% of the positive cases referred to CRCs, were judged not to be affected by one of the IEMs in the screening panel and were considered as false positive results. This percentage includes all those newborns who had no genetic variants or were harboring one variant (heterozygotes), those who had affected mothers, premature infants, those who had a nutritional deficiency, or those who were affected by other diseases compared to those considered in the panel.

Newborns with either the same pathogenic/likely pathogenic variant in both alleles (homozygotes) or two different variants pathogenic/likely pathogenic (compound heterozygotes) were considered affected.

**Figure 2:**
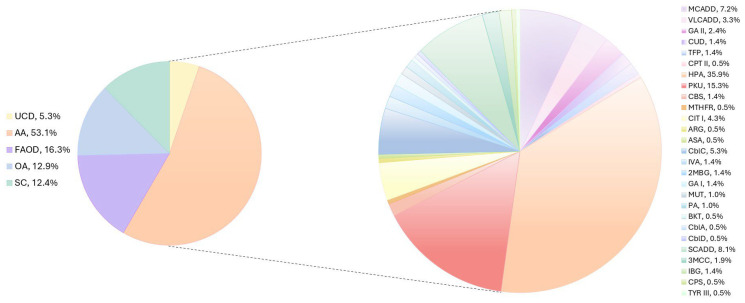
IEMs identified in Lombardy population via NBS. The pie chart on the left indicates the percentage of UCDs (yellow), AAs (peach), FAODs (violet), OAs (light blue), and SCs (emerald) determined in our cohort. The pie chart on the right displays the percentage of the different disorders calculated to the total of IEMs.

Out of 44 IEMs of the NBS panel, 27 types were diagnosed in the total of 209 patients: 111 newborns (53.1%) were affected by AAs, 11 (5.3%) by UCDs, 27 (12.9%) by OAs, 34 (16.3%) by FAODs, and 26 (12.4%) by SCs (Figure 2).

The average incidence for the IEMs in the investigated population was 1:1644 live births, with a male/female ratio of 110/99.

**Figure 3:**
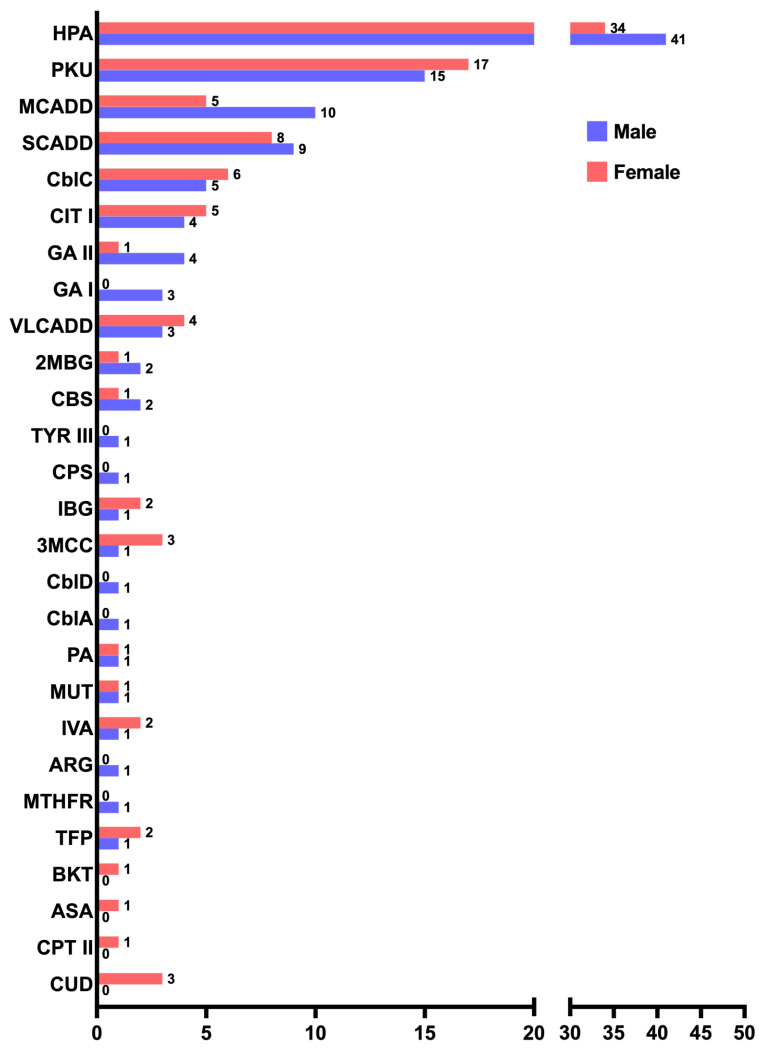
Distribution of males (violet) to females (orange) of the 27 IEMs identified in our cohort.

Although the total distribution of IEMs showed no differences between affected males and females (110/209 males, 53%; 99/209 females, 47%), when analyzing the IEMs individually, medium-chain acyl-CoA dehydrogenase deficiency (MCADD) was found to be two times more frequent in males than females in our cohort (Figure 3).

In the considered period of time, three false-negative cases were identified: one patient with methylenetetrahydrofolate reductase deficiency (MTHFR), one with glutaric acidemia type II (GA II), and one with hyperphenylalaninemia (HPA). The patient affected by MTHFR was a neonate born in 2016 (body weight of 2390 g), with a methionine concentration for the first DBS analyzed which fell within the first percentile range of the reference population and was dismissed. Notably, at that time, mandatory NBS had just been introduced and available reference population data were still limited.

The GA II patient was a severe preterm infant born at a gestational age of 27 weeks, with a birth weight of 780 g, who did not show any increase in disease-specific markers at NBS and was later diagnosed via whole exome sequencing.

The patient with HPA had Phe and Phe/Tyr ratio values above the cut-off for the first DBS, which then normalized on the second recalling for a DBS; therefore, he was not referred to the CRC and diagnosed subsequently.

**Figure 4:**
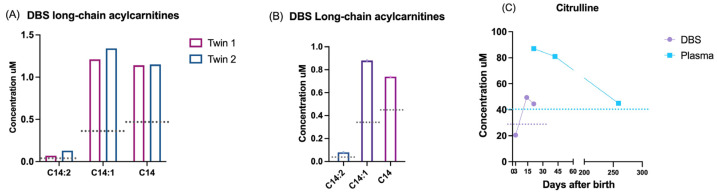
Exemplificative false positive cases. DBS biochemical results of the following: (**A**) acylcarnitines of two twins not mutated in *ACADVL* gene; (**B**) acylcarnitines of a newborn with a VUS having 25% of enzymatic activity; (**C**) DBS (violet) and plasma (blue) levels of citrulline in a newborn with negative genetic analysis. Dotted lines: 99° percentile cut-off referred for each marker.

Among all the false positives, we report four interesting cases: a couple of twins, with altered acylcarnitines C14:2, C14:1, and C14 and 50% of enzymatic activity of the very long-chain acyl-CoA dehydrogenase (VLCAD) protein, resulted in being genetically negative (Figure 4A); a newborn referred to a CRC for the persistent alteration of C14:2, C14:1, and C14 and the novel uncertain significance variant c.1291G>C (p.Asp431His) in the *ACADVL* gene showed an enzymatic activity of 25% (Figure 4B); one newborn with abnormal citrulline levels at birth resulted as negative to *SLC25A13* genetic analyses, and after six months of follow-up, the citrulline levels normalized, and no further genetic analyses were conducted (Figure 4C).

#### 3.1.1. Aminoacidemias (AAs)

In our cohort, four types of amino acid disorders were detected, with a total incidence of 1:3095. HPA were the most common diseases (75/209, 35.9%, 1:4580), followed by phenylketonuria (PKU) (32/209, 15.3%, 1:10,735), classical homocystinuria due to cystathionine beta synthase deficiency (CBS) (3/209, 1.4%, 1:114,502), and MTHFR defects (1/209, 0.5%, 1:343,507). One patient was found to be affected by Primapterinuria (OMIM #264070), a condition not included in the panel of screened diseases as it is asymptomatic, with transient hyperphenylalaninemia at birth; therefore, it is considered as a false positive at NBS.

#### 3.1.2. Urea Cycle Disorders (UCDs)

Three types of urea cycle disorders were detected in our cohort, with a total incidence of 1:31,228. The most frequently detected disease was Citrullinemia type I (CIT I) (9/209, 4.3%, 1:38,167), followed by Argininemia (ARG) (1/209, 0.5%, 1:343,507) and Argininosuccinic aciduria (ASA) (1/209, 0.5%, 1:343,507).

#### 3.1.3. Organic Acidemias (OAs)

Ten different organic acidemias were found in the studied population, with a total incidence of 1:12,722, in which there was combined methylmalonic aciduria and homocystinuria. The CblC type was the most representative (11/209, 5.3%, 1:31,228), followed by isovaleric aciduria (IVA) (3/209, 1.4%, 1:114,502), 2-methylbutyrylglycinuria (2MBG) (3/209, 1.4%, 1:114,502), glutaric acidemia type I (GA I) (3/209, 1.4%, 1:114,502), methylmalonic acidemia (MUT) (2/209, 1.0%, 1:171,754), propionic acidemia (PA) (2/209 1.0%, 1:171,754), betaketothyiolase deficiency (BKT) (1/209, 0.5%, 1:343,507), methylmalonic aciduria vitamin b12-responsive, due to a defect in the synthesis of adenosylcobalamin CblA type (1/209, 0.5%, 1:343,507), and methylmalonic acidemia and homocystinuria, CblD type (1/209, 0.5%, 1:343,507). One patient was found to be affected by ethylmalonic encephalopathy (EE) (OMIM #602473), which is a pathology not included in the mandatory panel of diseases for its poor prognosis; therefore, it was counted as a false positive.

#### 3.1.4. Fatty Acid Oxidation Disorders (FAODs)

Thirty-four patients were diagnosed with a fatty acid oxidation disorder, for a total of six diseases found, with a total incidence of 1:10,103, second to aminoacidemias. The most common disorders were MCADD (15/209, 7.2%, 1:22,900), VLCADD (7/209, 3.3%, 1:49,072), GA II (5/209, 2.4%, 1:68,701), trifunctional mitochondrial protein deficiency (TFP) (3/209, 1.4%, 1:114,502), carnitine uptake deficiency (CUD) (3/209, 1.4%, 1:114,502), and carnitine palmitoyltransferase 2 (CPT II) (1/209, 0.5%, 1:343,507). The three babies with TFP deficiency died in the first year of life.

#### 3.1.5. Secondary Conditions (SCs)

In the category of SCs, the most common disorder was short-chain acyl-CoA dehydrogenase deficiency (SCADD) (17/209, 8.1%, 1:20,206), followed by 3-methylcrotonyl-CoA carboxylase deficiency (3MCC) (4/209, 1.9%, 1:85,877), isobutyrylglycinuria (IBG) (3/209, 1.4%, 1:114,502) due to a deficiency of carbamoyl phosphate synthetase I (CPS) (1/209, 0.5%, 1:343,507), and type III tyrosinemia (TYR III) (1/209, 0.5%, 1:343,507).

## 4. Discussion

Since October 2016, the implementation of mandatory NBS in Italy, along with the introduction of mass spectrometry, has facilitated the early detection and treatment of rare IEMs in newborns. These conditions, which previously may have gone undiagnosed or were recognized only after irreversible clinical manifestations, can now be managed more effectively.

Lombardy is the region with the highest number of newborns in Italy, and its NBS panel is the largest in Europe [8]. Here, we report the data from the first five years of the expanded neonatal screening in Lombardy, during which 343,507 newborns were screened, leading to the identification of 209 affected children that have been treated consequently. The discrepancy between the total number of newborns and the number of DBSs analyzed is due to the limited participation in the first months of 2016, as the NBS program became mandatory by law in October 2016.

Studies from various countries revealed significant disparities in the reported incidence rates of IEMs. In some regions, higher rates of consanguinity can be associated with a variable rate of IEMs. Additionally, variations in the study methods and durations across different countries further complicate the understanding of overall incidence rates of IEMs [13,14,15].

In our cohort, we found a prevalence in AAs, followed by FAODs, OAs, and a small percentage of UCDs. In detail, AAs were found in 53.11% of the affected population, of which hyperphenylalaninemias ranked first, with an incidence of 1:4580. Among them, PKU represented 29%, with an incidence of 1:10,735, slightly higher than what has been reported by other screening centers in both other European states and worldwide [16,17]. In Lombardy, mild variants in the *PAH* gene are frequent, causing a phenotype that does not require dietary intervention except in phenylketonuria, confirming the evidence reported previously by Ruoppolo et al. [18,19].

FAODs were found in 16.27% of cases, and the predominant disease was MCADD, as expected [20,21]. This condition was the only IEM for which we observed a significant gender difference, as it was detected twice more in males compared to females. This trend could be influenced by the ethnic diversity, which is significantly higher in Lombardy compared to other Italian regions. In the initial 5 years of NBS, the incidence of MCADD (1:22,900) and VLCADD (1:49,072) was higher than expected [22]. This may be attributed to the fact that these patients were either not diagnosed or exhibited a mild phenotype with a low risk. In contrast to the findings reported in the literature [23,24], our cohort did not always demonstrate a consistent correlation between the genetic, enzymatic, and biochemical findings. In particular, within two reported VLCADD cases, despite the altered biochemical findings and the enzymatic function being at 50%, no alterations in the *ACADVL* gene were identified. Similarly, in the third case, a correlation was identified between the biochemical markers (e.g., increase in C14:0, C14:1, and C14:2) and residual enzymatic activity (25%) but the genetic analysis revealed only a variant of uncertain significance (VUS).

In all three cases, the increase in C14:1 level correlates with a reduced enzymatic activity, as reported also by Al Bandari et al. [25]. Thus, it emerges that the comprehensive global evaluation of biochemical and genetic data together with enzymatic activity is essential for the correct definition of the pathogenicity risk.

OAs, almost equivalent to FAODs, represent the third most frequent class of IEMs in the Lombardy cohort, affecting 12.92% of the affected population, with methylmalonic acidemias (CblA/C, CblB/D and MUT) (1:22,900) and IVA (1:114,502) being the two most common types. For these categories, the introduction of the 2TT was fundamental, as the increase in the primary marker value at NBS is not always pathognomonic of disease. Indeed, in some cases, the abnormal level of the analyzed biomarker can be a false positive result that unnecessarily leads to a newborn recall. The application of a 2TT able to identify free organic acids such as MMA and 3OH-PA or isobaric compounds such as IVA and 2MBC led to the reduction in the C3- and C5-false positive rates, respectively, and an increase in the C3- and C5-predictive positive values. As a result, the NBS specificity increased considerably, improving the diagnostic timing of affected newborns [10]. Many countries have introduced genetic tests such as the 2TT into their NBS programs, demonstrating significant benefits for certain conditions by excluding healthy carriers or pseudo-deficiencies. However, the longer turnaround times are still a limitation that does not allow these techniques to be used fully in clinical practice [26].

UCDs are present in only 5.26% of affected infants, and CIT I is the most frequent in our population, with an incidence of 1:42,938, as expected [27].

Three false negative cases were identified due to the lack of available population data. With increased experience, the expansion of the analyzed population, and the introduction of the 2TT, no more false negatives were reported. This underscores the need for reference population data adjusted for gestational age and birth weight to refine the cut-off values. This highlights the key role of the 2TT in either using a different method to improve the specificity of a biomarker or the detection of a different biomarker and highlights the importance of the ongoing basic research in identifying novel, more specific markers.

The false positive rate found in this study corresponds to 0.35% of the total newborns screened. It is important to underscore that if we calculate this percentage considering the newborns positive to NBS and referred to CRCs, it becomes even more significant: in fact, 85.24% (1208/1414) were not affected by any screened IEMs. This high percentage is likely due to the challenge in NBS of establishing a cut-off that prevents false negatives while simultaneously minimizing false positives. In fact, the strategy adopted by our laboratory during the first five years of the extended NBS was to adjust the cut-offs to enhance diagnostic sensitivity over specificity. Other aspects have contributed to this high rate, such as a low weight, birth stress, the need for neonatal intensive care, nutritional status, the presence of heterozygous variants, or the metabolic status of the mother. A mild to moderate increase in C3-acylcarnityne with hyperhomocysteinaemia is common in vitamin B12 or folate deficiencies as well as defects in riboflavin which can lead to GA II-like biochemical profiles that may contribute to false positives in newborn screening [28,29,30]. In order to better define the cut-offs, we are performing a correlation study between the biochemical data of false positives and their genetic findings to develop a strategy for refining this high percentage. Indeed, the greater harmonization of results across Italian screening centers would be highly desirable.

## 5. Conclusions

Here, we present data concerning the incidence and distribution of IEMs in the Lombardy population in the first five years of the mandatory NBS that contributed to the early diagnostic assessment of the affected newborns. The robustness of this study lies not only in the sample size, given that the Lombardy region has the highest birth rate in Italy, but also in the length of the observational period, which is crucial considering the rarity of the genetic diseases screened. Moreover, this study also highlights that further efforts are needed to reduce the number of false positives and minimize parental distress. In addition, in the forthcoming research, it would be important to integrate biochemical screening data with genetic analysis, enzymatic activity, clinical features, and follow-up, to highlight the novel diagnostic workflow that may more accurately describe pathological conditions or risk factors.

## Figures and Tables

**Table 1:** Panel of screened conditions in Lombardy region (Italy) according to ministerial decree of 13th October, biochemical, and 2nd-tier test (2TT) markers.

Abbreviation	OMIM	Gene	Biomarkers	2TT
***Aminoacidemias***				
PKU	261600	*PAH*	Phe↑, Phe/Tyr↑	
HPA	261600	*PAH*		
BIOPT	261630/261640	*QDPR/PTS*		
TYR I	276700	*FAH*	Suac↑, Tyr↑	
TYR II	276600	*TAT*	Tyr↑	
MSUD	248600	*BCKDHA*	Val↑, Xle↑	Ile/Leu/AlloIle
HCY	236200	*CBS*	Met↑	HCY
MTHFR	236250	*MTHFR*	Met↓	
***Urea cycle disorders***				
CIT I	215700	*ASS1*	Cit↑	
CIT II	605814	*SLC25A13*		
ASA	207900	*ASL*	Asa↑, Cit↑	
ARG	207800	*ARG1*	Arg↑	
***Organic acidemias***				
GA I	231670	*GCDH*	C5DC↑	*GA*↑, *3OHGA*↑
IVA	243500	*IVD*	C5↑	C5 isomers (IVA↑)
BKT	203750	*ACAT1*	C5:1↑, C5-OH↑	TYGLY↑
HMG	246450	*HMGCL*	C5OH↑, C6:DC↑	
PA	606054	*PCCA* *PCCB*	C3↑, C16:1OH↑	3OHPA↑, MCA↑, PAGLY↑
MUT	251000	*MMUT*		MMA↑, 3OHPA↑, MCA↑, PGLY↑
Cbl A	251100	*MMAA*		MMA↑
Cbl B	251110	*MMAB*		
Cbl C	277400	*MMACHC*	C3↑, C16:1OH↑, Met↓	MMA↑, HCY↑
Cbl D	277410	*MMADHC*		
2MBG	610006	*ACADSB*	C5↑	C5 isomers (2MBC↑)
MAL	606761	*MLYCD*	C3DC↑	
MCD	253270	*HLCS*	C5OH↑	
***Fatty acid oxidation disorders***				
CUD	212140	*SLC22A5*	C0↓	
CPT Ia	255120	*CPT1A*	C16↓, C18↓, C0/(C16 + C18)↑	
CACT	212138	*SLC25A20*	C0↓, C16↑, C18↑, C18:1↑	
CPT II	600650	*CPT2*	C16↑, C18:1↑, C2↓, C18↑, C18:2↑, (C16 + C18:1)/C2↑	
VLCADD	609575	*ACADVL*	C14:1↑, C14↑, C14:2↑, C14:1/C12:1↑, C14:1/C16↑	
TFP	609015	*HADHA* *HADHB*	C16OH↑, C18OH↑	
LCHAD	609016	*HADHA*		
MCADD	201450	*ACADM*	C6↑, C8↑, C10:1↑, C10↑	EXAGLY↑, SUBGLY↑
M/SCHAD	231530	*HADHSC*	C4OH↑	C4OH isobars (C4OH-S↑)
GA II/MADD	231680	*ETFA* *ETFB* *ETFDH*	C4↑, C5↑, C5DC↑, C6↑, C8↑, C10, C12↑, C14↑, C16↑, C18↑	GA↑, 2OHGA↑, EMA↑, C4-I↑, C4-B↑, IVAGLY↑, BUTGLY↑, EXAGLY↑, SUBGLY↑
***Secondary conditions***				
TYR III	276710	*HPD*	Tyr↑	
GNMT	606664	*GNMT*	Met↑	
MAT	250850	*MAT1A*		
SAHH	613752	*AHCY*	Met↑	
3MGCA	250950	*AUH*	C5-OH↑	
3MCC	210200	*MCC1*	C5-OH↑	
2M3HBA	300438	*HSD17B10*	C5:1↑ C5-OH↑	
IBG	611283	*ACAD8*	C4↑	C4-I↑, IBG↑
SCADD	201470	*ACADS*		EMA↑, C4-B↑, BUTGLY↑
CPS	237300	*CPS1*	Cit↓	

Xle = sum of isomers Ile/Leu/AlloIle/Pro-OH; HCY = homocysteine; GA = glutaric acid; 3OHGA = 3-hydroxyglutaric acid; TYGLY = tiglylglycine; 3OHPA = 3-hydroxypropionic acid; MCA = methylcitric acid; PGLY = propionyl glycine; 2MBC = 2-methylbutyryl carnitine; MMA = methylmalonic acid; EXAGLY = exanoyl glycine; SUBGLY = suberyl glycine; EMA = ethylmalonic acid; IBG = isobutyryl glycine; 2OHGA = 2-hydroxyglutaric acid; IVAGLY = isovaleryl glycine; BUTGLY = butyryl glycine; C4-I = isobutyryl carnitine; C4-B = butyrylcarnitine; ↑ = marker increase; ↓ = marker decrease.

**Table 2:** Marker cut-offs. Cut-offs here reported were established in 2018 based on a reference population of 134,000 newborn samples collected at 48–72 h of life from infants born after 37 weeks of gestation.

Markers	1° Percentile (μM)	99° Percentile (μM)	Mean	Median	Standard Deviation
***Amino acids***					
Orn	39.28	219.37	92.16	85.15	36.54
Val	69.57	245.4	134.31	129.3	36.9
Xle	82.73	250.11	145.97	141.56	35.34
Met	9.14	36.04	20.22	19.74	5.74
Arg	0.92	33.74	9.49	7.78	7.13
Cit	5.24	31.04	14.55	13.83	5.33
Asa	0	0.5	0.17	0.16	0.11
Phe	36.06	93.6	58.04	56.67	12.04
Tyr	42.38	233.9	58.04	56.67	12.04
Suac	0.23	1.7	99.45	91.84	39.11
***Acylcarnitines***					
C0	7.09	45.98	18.8	17.19	8.13
C2	10.17	56.11	25.19	23.53	9.54
C3	0.95	5.43	2.23	2.04	0.96
C4	0.09	0.86	0.26	0.23	0.13
C5:1	0	0.03	0.01	0.01	0.01
C5	0.05	0.29	0.11	0.1	0.06
C6	0.02	0.11	0.05	0.04	0.02
C8:1	0.01	0.1	0.03	0.03	0.02
C8	0.02	0.17	0.06	0.06	0.03
C10:2	0	0.02	0	0	0.01
C10:1	0.02	0.1	0.05	0.04	0.02
C10	0.03	0.27	0.1	0.09	0.05
C12:1	0.03	0.3	0.1	0.08	0.06
C12	0.04	0.37	0.13	0.11	0.07
C6DC	0.05	0.29	0.14	0.13	0.07
C14:2	0.01	0.05	0.02	0.02	0.01
C14:1	0.04	0.38	0.14	0.12	0.07
C14	0.11	0.52	0.25	0.24	0.09
C14OH	0.01	0.05	0.0187	0.020	0.0098
C16:1	0.1	0.53	0.27	0.26	0.09
C16	1.68	7.46	3.82	3.65	1.22
C16:1OH (C17)	0.02	0.09	0.05	0.04	0.01
C16OH	0.01	0.06	0.02	0.02	0.01
C18:2	0.06	0.48	0.17	0.15	0.09
C18:1	0.84	3.36	1.78	1.71	0.53
C18	0.51	2.28	1.14	1.08	0.37
C18:1OH	0.01	0.05	0.03	0.03	0.01
C18OH	0.01	0.03	0.02	0.02	0.01
C5OH (C4DC)	0.11	0.43	0.22	0.21	0.07
C3DC (C4OH)	0.07	0.5	0.22	0.21	0.09
C5DC (C6OH)	0.06	0.29	0.14	0.13	0.06

## Data Availability

Data are unavailable due to privacy.

## Abbreviations

The following abbreviations are used in this manuscript:

NBS	Newborn Screening
IEMs	Inborn Errors of Metabolism
DBS	Dried Blood Spot
CRC	Clinical Reference Center
AAs	Aminoacidemias
UCDs	Urea Cycle Defects
OAs	Organic Acidemias
FAODs	Fatty Acid Oxidation Disorders
SCs	Secondary Conditions
2TT	Second-Tier Test
plACs	Plasma Acylcarnitines
plAAs	Plasma Aminoacids
GC-MS	Gas Chromatography–Mass Spectrometry
CLIRs	Collaborative Laboratory Integrated Reports
MCADD	Medium-Chain Acyl-CoA Dehydrogenase Deficiency
MTHFR	Methylenetetrahydrofolate Reductase
GA II	Glutaric Acidemia type II
HPA	Hyperphenylalaninemia
VLCADD	Very Long-Chain Acyl-Coa Dehydrogenase Deficiency
PKU	Phenylketonuria
CBS	Classical homocystinuria due to cystathionine beta synthase
CIT I	Citrullinemia Type I
ARG	Argininemia
ASA	Argininosuccinic aciduria
IVA	Isovaleric Aciduria
2MBG	2-Methylbutyrylglycinuria
GA I	Glutaric Acidemia type I
MUT	Methylmalonic Acidemia
PA	Propionic Acidemia
BKT	Betaketothyiolase Deficiency
EE	Ethylmalonic Encephalopathy
TFP	Trifunctional Mitochondrial Protein Deficiency
CUD	Carnitine Uptake Deficiency
CPT II	Carnitine Palmitoyltransferase 2
SCADD	Short Chain Acyl-Coa Dehydrogenase Deficiency
3MCC	3-Methylcrotonyl-CoA Carboxylase deficiency
IBG	Isobutyrylglycinuria
CPS	Carbamoyl Phosphate Synthetase I
TYR III	Type III Tyrosinemia
VUS	Variant of Uncertain Significance
