# Correction: Berardo et al. Expanded Newborn Screening in Italy: The First Report of Lombardy Region. *Int. J. Neonatal Screen.* 2025, *11*, 31

**DOI:** 10.3390/ijns11030057

**Published:** 2025-07-22

**Authors:** Clarissa Berardo, Alessandra Vasco, Alessia Mauri, Simona Lucchi, Laura Cappelletti, Laura Saielli, Manuela Rizzetto, Davide Biganzoli, Cristina Montrasio, Diana Postorivo, Michela Perrone Donnorso, Elisa Pratiffi, Andrea Meta, Stephana Carelli, Alessandro Amorosi, Sabrina Paci, Graziella Cefalo, Francesca Furlan, Francesca Menni, Serena Gasperini, Viola Crescitelli, Giuseppe Banderali, Gianvincenzo Zuccotti, Luisella Alberti, Cristina Cereda

**Affiliations:** 1 Pediatric Research Center “Romeo ed Enrica Invernizzi”, Department of Biomedical and Clinical Sciences, University of Milan, 20157 Milan, Italy; clarissa.berardo@unimi.it (C.B.); alessia.mauri@unimi.it (A.M.); stephana.carelli@guest.unimi.it (S.C.); gianvincenzo.zuccotti@unimi.it (G.Z.); 2 Center of Functional Genomics and Rare Diseases, Department of Pediatrics, Buzzi Children’s Hospital, 20154 Milan, Italy; alessandra.vasco@asst-fbf-sacco.it (A.V.); simona.lucchi@asst-fbf-sacco.it (S.L.); cappelletti.laura@asst-fbf-sacco.it (L.C.); laura.saielli@asst-fbf-sacco.it (L.S.); manuela.rizzetto@asst-fbf-sacco.it (M.R.); davide.biganzoli@unimi.it (D.B.); cristina.montrasio@asst-fbf-sacco.it (C.M.); diana.postorivo@asst-fbf-sacco.it (D.P.); michelaperronedonnorso@gaslini.org (M.P.D.); elisa.pratiffi@asst-fbf-sacco.it (E.P.); andrea.meta@asst-fbf-sacco.it (A.M.); luisella.alberti@asst-fbf-sacco.it (L.A.); 3 Welfare General Directorate, Regione Lombardia, Palazzo Lombardia, Piazza Città di Lombardia, 1, 20124 Milan, Italy; alessandro_amorosi@regione.lombardia.it; 4 Clinical Department of Pediatrics, ASST Santi Paolo e Carlo Hospital, University of Milan, 20142 Milan, Italy; sabrina.paci@asst-santipaolocarlo.it (S.P.); graziella.cefalo@asst-santipaolocarlo.it (G.C.); giuseppe.banderali@asst-santipaolocarlo.it (G.B.); 5 Pediatric Intensive Care Unit, Fondazione IRCCS Cà Granda Ospedale Maggiore Policlinico, 20122 Milan, Italy; francesca.furlan@policlinico.mi.it (F.F.); francesca.menni@policlinico.mi.it (F.M.); 6 Pediatric Rare Diseases Unit, Fondazione IRCCS San Gerardo dei Tintori, 20900 Monza, Italy; serena.gasperini@irccs-sangerardo.it (S.G.); viola.crescitelli@irccs-sangerardo.it (V.C.); 7 Department of Pediatrics, Buzzi Children’s Hospital, 20154 Milan, Italy

- **Addition of an author**

Michela Perrone Donnorso was not included as an author in the original publication [1]. Her name should be added between Diana Postorivo and Elisa Pratiffi. Her affiliation should be listed as Affiliation 2: “Center of Functional Genomics and Rare Diseases, Department of Pediatrics, Buzzi Children’s Hospital, 20154 Milan, Italy”. A note was also added to reflect her current address: “Pediatric Clinic, Endocrinology, Laboratory for the Study of Inborn Errors of Metabolism, Department of Neuroscience, Rehabilitation, Ophthalmology, Genetics, Maternal and Child Health, University of Genova, 16100 Genova, Italy”. The Author Contributions statement was also corrected accordingly.

**Author Contributions:** Conceptualization, C.B., A.V. and A.M. (Alessia Mauri); methodology, M.P.D., E.P. and A.M. (Andrea Meta); validation, M.R., C.M., D.P., S.P., G.C., G.B., F.F., F.M., S.G. and V.C.; formal analysis, D.B.; investigation, S.L., L.C., L.S. and L.A.; writing—original draft preparation, C.B., A.V. and A.M. (Alessia Mauri); writing—review and editing, S.C. and C.C.; supervision, G.Z., A.A. and C.C. All authors have read and agreed to the published version of the manuscript.

- **Text Correction**

In addition, patent information was not included in the text. A correction has been made to the Materials and Methods, DBS Analysis, Second-Tier Tests, Section 2.3.

The 2TT result confirms or overrules the primary screening result. The use of the 2TT can identify the same target as primary screening but with improved specificity due to the separation from isomers or interfering substances, or it can screen for another diagnostic marker not included in the first-tier screening [10]. Patent: Perrone Donnorso, M., Cassanello, M., Cereda, C. A method and a kit for multiplexed 2nd tier test application in Newborn Screening. Pending Patent application n. 102024000022824, 14 October 2024.

The authors state that the scientific conclusions are unaffected. This correction was approved by the Academic Editor. The original publication has also been updated.
